# Adaptive metabolic strategies of intracellular bacterial pathogens

**DOI:** 10.3389/fmicb.2026.1783835

**Published:** 2026-03-31

**Authors:** Tripti Nair, Eric H. Rosenn, Biplab Singha, Shagun Shukla, Sharad Vashisht, Aditya Upadhyay, Murugesh Padmanarayana, Vijay Soni

**Affiliations:** 1 Leonard Davis School of Gerontology, University of Southern California, Los Angeles, , CA, United States; 2 Departments of Biomedical Engineering, New York University Tandon School of Engineering, Brooklyn, NY, United States; 3 Department of Medicine and Biomedical Science, Cedars-Sinai Medical Center, Los Angeles, CA, United States; 4 Department of Neurology, Feinberg School of Medicine, Northwestern University, Chicago, IL, United States; 5 Humankine, New Product Development, Proteintech Group Inc., Rosemont, IL, United States; 6 Department of Cell and Developmental Biology, Graduate School of Medical Sciences, Weill Cornell Medicine, New York, NY, United States; 7 Orthopedic Soft Tissue Research Program, Hospital for Special Surgery, New York, NY, United States; 8 Department of Biochemistry, Beckman Center for Molecular and Genetic Medicine, Stanford University, Palo Alto, CA, United States; 9 Division of Infectious Diseases, Weill Department of Medicine, Weill Cornell Medicine, New York, NY, United States

**Keywords:** antimicrobial resistance (AMR), bipartite metabolism, host-directed therapies (HDTs), host-pathogen interactions, immunometabolic signaling, intracellular bacterial pathogens, metabolic reprogramming, metabolomics

## Abstract

Intracellular bacterial pathogens have evolved sophisticated metabolic strategies to persist and replicate within the hostile intracellular environments of their hosts. By leveraging their metabolic plasticity, these pathogens dynamically modulate host metabolic processes in response to immunological, environmental, and pharmacological stressors. This review examines the diverse metabolic adaptations employed by intracellular pathogens, including nutrient acquisition, modulation of host metabolism, and stress-induced metabolic shifts that contribute to persistence and virulence. Emphasis is placed on how distinct intracellular niches- such as vacuoles and the cytosol- shape pathogen metabolism, and how bipartite metabolic strategies enable pathogens to balance energy production with biosynthetic demands. Species-specific adaptations in representative pathogens, including *Listeria monocytogenes*, *Legionella pneumophila*, *Shigella flexneri*, and *Chlamydia trachomatis*, are analyzed, with a focus on mechanisms of metabolic reprogramming (the alteration of cellular metabolic pathways in response to environmental cues, such as infection or stress, which allows the pathogen to adapt its metabolic state to support survival, replication, and virulence within the host), stress tolerance (refers to a pathogen’s ability to survive and function under harsh environmental conditions, such as oxidative stress, nutrient scarcity, and antimicrobial exposure), and lifecycle transitions (refer to the changes in a pathogen’s developmental or replication stages, such as switching from active growth to a dormant or persistent state during infection). Finally, the review considers how these metabolic strategies intersect with antimicrobial resistance and highlights the potential of targeting host-pathogen metabolic interactions for the development of novel interventions, including host-directed therapies (HDTs).

## 1 Introduction

**FIGURE 1:**
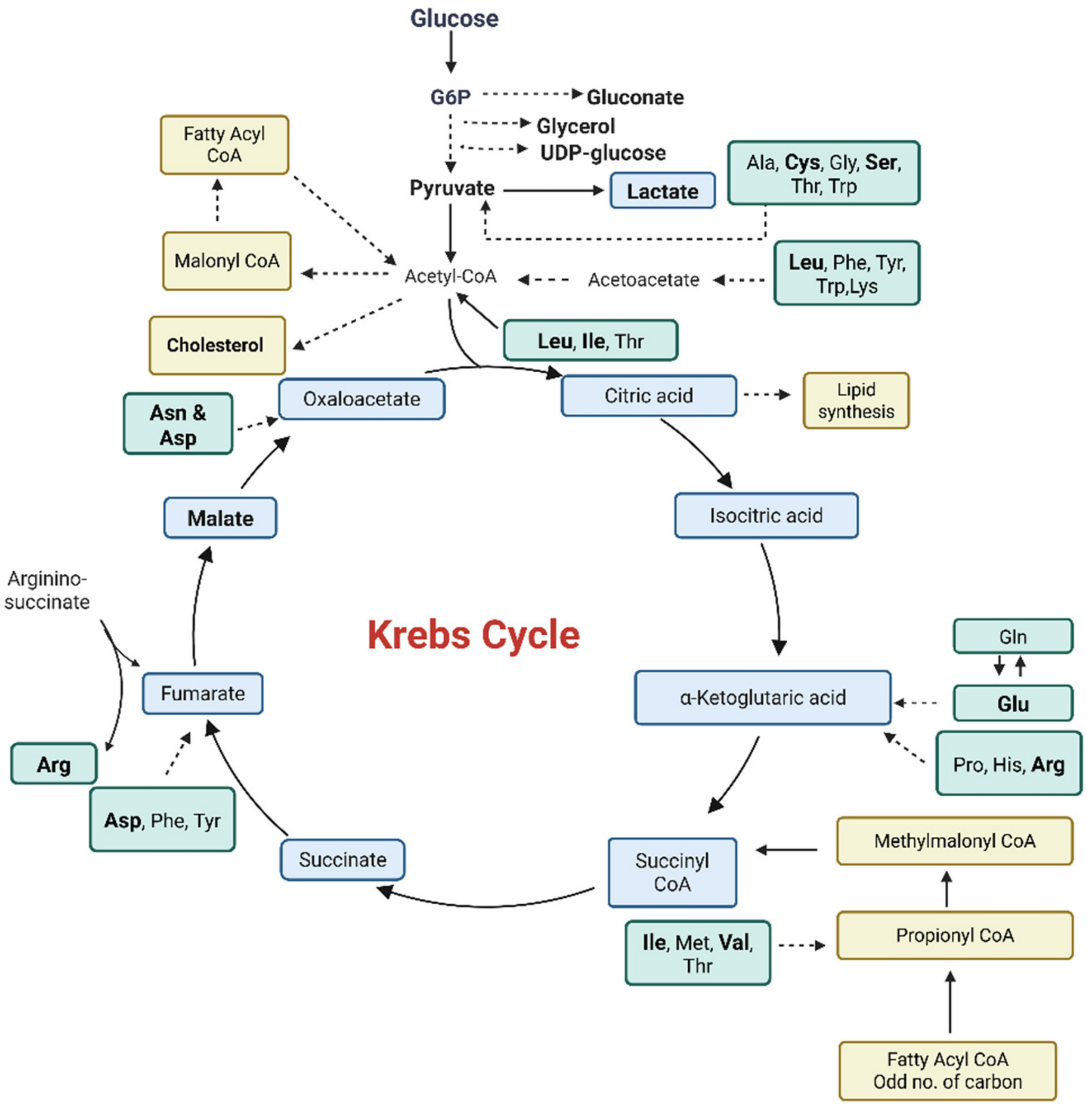
The diagram summarizes central metabolic pathways in bacterial cells that are commonly modulated during intracellular infection. Metabolites and pathways shown in bold indicate metabolic nodes reported to have increased activity or utilization during intracellular growth compared with extracellular conditions across multiple intracellular pathogens. These pathways support nutrient acquisition, biosynthesis, virulence, and stress adaptation. “Infection stage” refers to the intracellular phase of the bacterial lifecycle within host cells, while “resistance” denotes metabolic adaptations that promote bacterial survival under host-imposed stresses. The schematic represents a generalized overview derived from multiple intracellular bacterial pathogens rather than a single species. Image created using Biorender.com.

Bacterial pathogens have developed distinctive metabolic adaptations that support their survival by scavenging nutrients, salvaging metabolites, and reprogramming both host and microbial metabolic pathways to overcome hostile environments. One key strategy employed by intracellular pathogens is bipartite metabolism, where nutrient acquisition is divided into two functional networks: one dedicated to energy production (catabolism) and the other to biosynthesis (anabolism). This metabolic division allows pathogens to balance the need for energy production while meeting the biosynthetic demands of replication within the host cell. These changes include modifications in central carbon metabolism (Figure 1), amino acid biosynthesis, lipid production, nucleotide salvage, and many other biochemical adaptations that enhance bacterial fitness. Metabolic flexibility has significantly influenced the evolutionary dynamics between pathogens and their human hosts (Moye et al., 2014). For instance, *Streptococcus mutans* manipulates host carbohydrate metabolism to support its growth, while *Mycobacterium tuberculosis* relies on host lipid metabolism to maintain chronic infection (Gago et al., 2018; Singha et al., 2024). Pathogens can also induce specific host cell phenotypes, such as lipid accumulation in macrophages, or directly extract complex carbohydrates from host tissues. For example, metabolomics analyses of *Salmonella enterica* infected human macrophages have revealed increased glucose content, higher rates of glucose uptake and glycolysis, and decreased oxidative phosphorylation (Wang et al., 2021; Figure 1).

Intracellular bacterial pathogens are broadly classified based on their dependence on the intracellular niche. Facultative intracellular bacteria, such as *Salmonella*, *Mycobacterium*, *Brucella*, and *Legionella*, can replicate both within host cells and in extracellular environments, including natural reservoirs and laboratory media (Eisenreich et al., 2015). In contrast, obligate intracellular pathogens, including *Coxiella* and *Chlamydia*, are restricted to replication within host cells and cannot complete their life cycle extracellularly. Within host cells, these bacteria adopt distinct intracellular lifestyles—some remain enclosed within modified pathogen-containing vacuoles (PCVs), while others, such as *Shigella*, *Listeria*, *Rickettsia*, and *Francisella*, escape into the cytosol to replicate.

The dynamic interactions between these bacteria and their intracellular environments involve extensive remodeling of both host and pathogen metabolism. While many aspects of these interactions remain incompletely understood, advances in analytical techniques, including comparative transcriptomics, proteomics, metabolomics, C^13^-isotopologue profiling, and metabolic flux analysis using NMR, mass spectrometry, secondary-ion mass spectrometry, and Raman spectroscopy- have begun to illuminate the metabolic strategies employed during intracellular infection (Eisenreich et al., 2015; López-Jiménez and Mostowy, 2021).

In this article, we review various metabolic strategies employed by intracellular pathogens and elucidate the signaling pathways involved in sensing and adjusting to environmental changes. Adaptations throughout the bacterial lifecycle, such as bipartite metabolism and shifts in intracellular location, will be explored. It gives a concise overview of the metabolic and lifecycle adaptations displayed by several key pathogens, including *Listeria monocytogenes*, *Legionella pneumophila*, *Shigella flexneri*, and *Chlamydia trachomatis*. Finally, the role of metabolic adaptations in the development of antimicrobial resistance (AMR) and potential treatment strategies will be discussed. Ultimately, a deeper understanding of metabolic reprogramming will enhance our ability to address the molecular mechanisms underpinning AMR and support the development of effective treatments, including host-directed therapeutics (HDTs).

## 2 Host-pathogen metabolic integration during intracellular infection

### 2.1 Immunometabolic reprogramming

Upon infection, host immune cells, particularly macrophages, undergo profound metabolic reprogramming that reshapes infection outcomes. Activation of Pattern-recognition receptor (PRRs)—innate immune receptors that detect conserved microbial molecules, particularly via Toll-like receptors (TLRs), initiates a switch from mitochondrial oxidative phosphorylation to aerobic glycolysis (Rosenberg et al., 2022). This metabolic shift fuels biosynthetic demands essential for cytokine production, antimicrobial responses, and innate immune activation (Troha and Ayres, 2020). Concurrently, the host restricts key nutrients such as iron and amino acids to suppress microbial growth through nutritional immunity (Sprenger et al., 2018). However, intracellular pathogens have evolved strategies to exploit these metabolic changes for replication and persistence.

Pathogens directly subvert host metabolism to enhance intracellular survival. *Listeria monocytogenes* upregulates host hexokinase II to increase glucose-6-phosphate availability (Chico-Calero et al., 2002), while *Shigella flexneri* suppresses host p53 in epithelial cells to boost glucose uptake and pyruvate capture (Bergounioux et al., 2012). *Salmonella enterica* manipulates host vesicular trafficking to direct nutrient-rich vesicles toward pathogen-containing vacuoles (Eisenreich et al., 2015). These adaptations illustrate how intracellular bacteria remodel host metabolic pathways to optimize their intracellular niche (Amer and Swanson, 2005; Price et al., 2011; Wieland et al., 2005).

In addition to direct pathogen-driven metabolic reprogramming, infection also triggers inflammatory signaling in macrophages that broadly reshapes host cellular metabolism. Inflammatory signaling disrupts the host tricarboxylic acid (TCA) cycle by nitric oxide (NO) mediated inhibition of aconitase and repression of isocitrate dehydrogenase (IDH1), resulting in the accumulation of citrate and cis-aconitate (Choi et al., 2021). Citrate fuels fatty acid synthesis and the production of itaconate via immune-responsive gene 1 (IRG1) (Zeng et al., 2023). Itaconate exhibits antimicrobial activity within macrophage polarization (MP) phagosomes by inhibiting bacterial enzymes such as isocitrate lyase and succinate dehydrogenase (Priya et al., 2025). Concurrently, succinate accumulation stabilizes hypoxia-inducible factor 1α (HIF-1α), promoting glycolysis and interleukin-1β (IL-1β) production to trigger MP polarization (Mills et al., 2016; Tannahill et al., 2013). Aerobic glycolysis also promotes the formation of lipid droplets (LDs)- single-membrane organelles consisting mainly of triacylglycerol and cholesterol that serve as immunometabolic signaling hubs- by converting excess citrate into fatty-acyl chains used for triacylglycerol synthesis (Walther and Farese, 2012). LDs containing host defense proteins such as cathelicidin can detach from mitochondria to interact with bacteria and enhance phagocytosis, as well as regulate proinflammatory response activation (Bosch et al., 2020). Pathogens such as *Salmonella typhi* (causing typhoid fever) exploit this altered metabolic landscape by inducing glycolysis while suppressing oxidative phosphorylation to enhance glucose availability. Meanwhile, *Salmonella enterica serovar Typhimurium* a major cause of gastroenteritis, manipulates host metabolism through the type III secretion system effector SopE2. SopE2 acts as a guanine nucleotide exchange factor that activates host Rho-family GTPases such as Cdc42, leading to cytoskeletal rearrangements and metabolic reprogramming. This process promotes the accumulation of glycolytic intermediates such as 3-phosphoglycerate and alters host serine metabolism, thereby providing metabolic substrates that support bacterial intracellular replication (Eriksson et al., 2003; Wang et al., 2021).

In addition to glycolytic remodeling, mitochondria are critical targets for pathogen manipulation. Mitochondrial reactive oxygen species amplify antimicrobial signaling, whereas lactate generated via glycolysis triggers inflammasome activation, linking cellular metabolism to innate immunity (Rosenberg et al., 2022). Pathogens such as *Legionella pneumophila* actively disrupt mitochondrial respiration through type IV secretion effectors, disrupting oxidative metabolism and increasing bacterial persistence (Maurice and Sadikot, 2023). Similar strategies have been reported in other intracellular pathogens; for example, *Mycobacterium tuberculosis* can modulate host mitochondrial metabolism to reduce oxidative stress and support intracellular persistence, while *Chlamydia trachomatis* alters host energy metabolism to maintain a favorable niche for replication (Mohareer et al., 2020; Rother et al., 2019).

As infection progresses, macrophages transition toward an anti-inflammatory state orchestrated by nuclear factor erythroid 2-related factor 2 (NRF2) and peroxisome proliferator-activated receptor gamma (PPARγ). This reprogramming involves a shift from glycolysis to fatty acid oxidation and glutaminolysis that restores mitochondrial oxidative phosphorylation and facilitates the resolution of inflammation (O’Neill and Artyomov, 2019; Van den Bossche et al., 2016). Epigenetic modifications mediated by metabolites such as α-ketoglutarate and lactate contribute to this transition, reinforcing anti-inflammatory gene expression. Altogether, these dynamic shifts in host metabolism create evolving intracellular niches that bacterial pathogens sense and exploit to optimize nutrient acquisition and evade immune defenses.

### 2.2 Nutrient adaptation strategies

Intracellular pathogens have evolved common strategies to overcome host metabolic restrictions and satisfy their unique nutritional demands within the host cell (Table 1). Despite differences in lifestyle, obligate intracellular bacteria such as *Chlamydia*, *Coxiella*, *Anaplasma*, *Ehrlichia*, and *Rickettsia* all converge on targeting host cholesterol, both as a source of membrane lipids and as a means to manipulate host signaling and vesicular trafficking (Samanta et al., 2017). In many cases, pathogens also exploit cholesterol-rich microdomains, or lipid rafts, which serve as organizational platforms for host phagocytic signaling pathways and facilitate access to intracellular niches (Samanta et al., 2017).

**TABLE 1:** Metabolic changes in bacterial pathogens.

Nutrient class	Impact	Nutrient source	Bacterial species	Influence on intracellular methods	References
Carbohydrates and polyols	Virulence gene expression	Glycerol	*L. monocytogenes*	Phagosomal escape.	Mertins et al., 2007
	Energy source	Glucose-6-phosphate	*L. monocytogenes*	Intracellular proliferation	Grubmüller et al., 2014
		Glycerol			
	Carbon source	Glycerol	*F. tularensis*		Ziveri et al., 2017
		Glycerol	Enteroinvasive *E. coli*, S. enterica, S. flexneri		Eisenreich et al., 2015; Eriksson et al., 2003
		Glucose-6-phosphate	*L. monocytogenes*, Enteroinvasive *E. coli*, *S.* Typhimurium, *S. flexneri*, *C. trachomatis*		Eisenreich et al., 2015; Grubmüller et al., 2014
		Glucose	*S. enterica*, *S. flexneri* *S.* Typhimurium, Enteroinvasive *E. coli*		Eisenreich et al., 2015; Eriksson et al., 2003
		Gluconate	*S.* Typhimurium		Eisenreich et al., 2015
		Lactate	*S. flexneri*, Enteroinvasive *E. coli*, *R. prowazekii*		
		Pyruvate	*R. prowazekii*		
		Malate			
		Uridine 5’-diphosphoglucose			
Amino acids	Virulence and morphological differentiation	BCAA glutamine	*L. monocytogenes*	Phagosomal escape	Lobel et al., 2015; Haber et al., 2017
		Arginine	*L. pneumophila*	Intracellular proliferation	Hovel-Miner et al., 2010
	Protein biosynthesis, nitrogen assimilation	Asparagine	*L. monocytogenes*, *M. tuberculosis*	Intracellular proliferation, modification of phagosomal pH	Grubmüller et al., 2014; Gouzy et al., 2014
	Oxidative stress resistance	Glutamate	*F. tularensis*	Phagosomal escape	Ramond et al., 2014
	Energy source	Asparagine	*F. tularensis*	Intracellular proliferation	Gesbert et al., 2014
	Phagosomal escape	Glutamate	*F. tularensis*, *C. trachomatis* *C. trachomatis*		Eisenreich et al., 2015; Ramond et al., 2014
		Isoleucine	*F. tularensis*	Phagosomal escape	Gesbert et al., 2014
		Serine	*L. pneumophila*		Gillmaier et al., 2016; Häuslein et al., 2016
		Aspartate	*C. trachomatis*		Eisenreich et al., 2015
		Arginine	*F. tularensis*		Ramond et al., 2015
	Sulfur source	Isoleucine	*F. tularensis*	Intracellular proliferation	Gesbert et al., 2015
		Cysteine/glutathione			Meibom and Charbit, 2010
Lipids	Carbon, nitrogen and phosphorus source	Sphingomyelin	*M. tuberculosis*	Intraphagosomal replication	Pandey and Sassetti, 2008; Speer et al., 2015
	Energy source	Cholesterol			
Vitamins	Cofactor activity	Biotin	*F. tularensis*	Phagosomal escape	Napier et al., 2012
		Riboflavin	*M. marinum* *H. capsulatum*	Intracellular proliferation	Yu et al., 2011; Garfoot and Rappleye, 2016; O’Riordan et al., 2003
		Lipoic acid	*L. monocytogenes*		
Trace elements	Cofactor activity	Iron	*S.* Typhimurium, *S. aureus*, *Y. pestis*, *C. jejuni* *Shigella flexneri*, *Mycobacterium tuberculosis*, *Legionella pneumophila*	Replication, transcription, metabolism and energy generation	Sprenger et al., 2018; Passalacqua et al., 2016
		Manganese	*S. aureus*	Carbon metabolism and oxidative stress resistance	
		Zinc	*E. coli*, *S.* Typhimurium, *A. baumannii*, *M. tuberculosis.*	Structural and catalytic roles among a large number of proteins	
		Copper	*M. tuberculosis*, *E. coli*, *P. aeruginosa*	Used by metalloenzymes of ETC reactions	

To survive within host compartments, intracellular pathogens must adapt to limited access to essential nutrients. Glucose, amino acids, nucleotides, and lipids are all critical for bacterial survival but are tightly regulated by host cell metabolism. To overcome this, pathogens deploy diverse strategies:

1. *Mycobacterium tuberculosis* exploits host lipid droplets and shifts to lipid catabolism during chronic infection (Gago et al., 2018; Lee et al., 2013; van der Klugt et al., 2024). This shift is supported by the induction of enzymes involved in β-oxidation and the glyoxylate shunt, which bypasses carbon loss and facilitates biomass generation under nutrient-limited conditions (Table 1).
2. *Salmonella enterica* induces host arginine transporters to increase arginine availability within the *Salmonella*-containing vacuole (SCV) (Kehl et al., 2020; Margolis et al., 2023). Arginine metabolism is not only central to bacterial growth but also modulates host nitric oxide production, influencing immune outcomes (Table 1).
3. *Chlamydia trachomatis* and *Legionella pneumophila* co-opt host solute carrier (SLC) transporters to import nutrients directly into pathogen-containing vacuoles (Best and Abu Kwaik, 2019; Di Pietro et al., 2019). In the case of *Chlamydia*, the SLC35D2 transporter delivers UDP-glucose, which is used to synthesize glycogen stores within the inclusion (Gehre et al., 2016; Table 1).

### 2.3 Iron and micronutrient scavenging

Iron is a critical cofactor for both host and pathogen metabolism. Hosts limit pathogen access to iron via sequestration in ferritin, induction of hepcidin, and downregulation of transferrin receptors, while pathogens counteract these defenses by secreting siderophores or acquiring iron through host lysosomes (Ullah and Lang, 2023; Table 1). *Shigella flexneri* upregulates multiple iron uptake systems including FeoB, SitABCD, and FhuA, and the Suf operon under iron-limiting conditions (Runyen-Janecky et al., 2003). *M. tuberculosis* expresses IrtAB and other specialized Fe transporters that function under oxidative and hypoxic stress (Arnold et al., 2020). *L. pneumophila* increases expression of iron acquisition genes in response to low cytoplasmic iron and uses ankyrin-repeat effectors to co-opt host iron transport machinery (Cianciotto, 2015). This tug-of-war over micronutrients exemplifies the metabolic competition that underlies host-pathogen interactions.

Micronutrients such as zinc, manganese, and copper also play important roles in host defense and pathogen survival (Table 1). For example, *Salmonella* encodes ZupT and SitABCD systems for zinc and manganese uptake (Cerasi et al., 2014), while *Listeria* relies on Mn^2+^ acquisition to support superoxide dismutase (SOD) function under oxidative stress (Mains et al., 2021). These micronutrients are also linked to the regulation of virulence genes, connecting the pathogen’s metabolic state to its pathogenic potential.

### 2.4 Metabolic adaptations to intracellular stressors

Bacteria are exposed to various biotic and abiotic stressors during pathogenesis, including temperature fluctuations, acidic environments, nutrient scarcity, and hypoxic conditions. Bacteria with successful survival strategies evolve increased virulence and tolerance to different therapeutic challenges. Below, we highlight key metabolic stress responses that facilitate bacterial adaptation and persistence.

*Acidic pH environments:*

Pathogens such as *Salmonella enterica*, *Mycobacterium*, and *Helicobacter pylori* confront acidic conditions while traversing compartments such as the gastrointestinal tract and lysosomes (Monack, 2013). To survive, bacteria employ two-component systems (TCS) such as PmrB/PmrA, PhoQ/PhoP, EvgS/EvgA, and EnvZ/OmpR, which sense acidic pH and initiate adaptive signaling cascades. Transcriptional regulators such as the Ferric uptake regulator (Fur) (Chen et al., 2020), and alternative sigma factors σ*^S^* and σ*^E^* also play crucial roles in modifying gene expression in response to pH-induced stress (Muller et al., 2009). In *Salmonella*, regulators PhoP, OmpR, and σS also orchestrate virulence gene expression, linking pH adaptation to pathogenicity (Fang et al., 1992). *M. tuberculosis* exhibits acid-induced growth arrest and metabolic reprogramming, shifting toward anaplerotic pathways and carbonic anhydrase activity to maintain pH homeostasis and energy production (Baker and Abramovitch, 2018). Recent studies further show that under acidic conditions *M. tuberculosis* preferentially utilizes host-derived lipids as carbon sources, enabling bacterial growth and persistence in low-pH environments. Carbonic Anhydrase (CA) catalyzes the reversible conversion of CO_2_ and H_2_O into HCO_3_^–^ and H^+^. This reaction buffers intracellular pH and supports pH-sensitive metabolic processes such as fatty acid oxidation and proteolysis (Capasso and Supuran, 2024; Capasso et al., 2012; Capasso, 2023; Del Prete et al., 2020; Merchant and Helmann, 2012; Supuran and Capasso, 2016; Supuran, 2008; van Elsas et al., 2011). The importance of this pathway is underscored by the reliance of *H. pylori* on urease and carbonic anhydrase to colonize the acidic gastric mucosa (Benito et al., 2023; Modak et al., 2019; Ronci et al., 2019). *M. tuberculosis* and *Salmonella* require CA for proliferation, survival, and pathogenesis, thus marking CA as a promising therapeutic target (Carta et al., 2009; Ceruso et al., 2014; Kohler et al., 2017; Nishimori et al., 2011; Rollenhagen and Bumann, 2006; Singh and Supuran, 2014; Vullo et al., 2011).

*Hypoxic conditions:*

Low oxygen availability within host tissues forces pathogens to alter respiratory strategies. For instance, *E. coli* switches to nitrate respiration under hypoxia (Jones et al., 2007), while *S. aureus* activates the SrrAB system that adjusts cellular processes and resource allocation (Butrico and Cassat, 2020). *M. tuberculosis* relies on DosR and PhoP to regulate genes involved in redox and nitrogen balance, enabling persistence under hypoxic stress (Singh et al., 2020). Hypoxia also promotes dormancy and phenotypic tolerance to antibiotics in many pathogens. Adaptation involves not only changes in terminal electron acceptors but also induction of high-affinity cytochrome oxidases and alternative fermentative pathways (Banerjee et al., 2023). These mechanisms are increasingly recognized as contributors to latent infections and treatment failures.

*Nutrient limitation:*

Pathogens sense nutrient depletion through global regulators such as (p)ppGpp, CodY, FruR/Cra, and CsrA, which reprogram metabolism and enhance virulence (Sun and O’Riordan, 2013). For instance, (p)ppGpp accumulation during amino acid starvation induces expression of virulence genes in *F. tularensis* and *V. cholerae* (Dalebroux et al., 2010). CodY integrates amino acid sensing with regulation of key metabolic and virulence genes in Gram-positive pathogens like *Listeria* and *S. aureus* (Pellegrini et al., 2022), while in *Shigella* the Cra/FruR system modulates carbon utilization based on metabolite levels (Gore and Payne, 2010). These central nutrient-sensing factors are hubs of metabolic networks and are attractive targets for therapeutic intervention.

## 3 Intracellular metabolic landscapes: pathogen adaptation to host microenvironments

### 3.1 Metabolic strategies in vacuolar and cytosolic niches

Intracellular pathogens adopt distinct metabolic strategies depending on their location within host cells. Those residing in pathogen-containing vacuoles (PCVs), such as *Salmonella enterica*, *Legionella pneumophila*, *Brucella* spp., and *Chlamydia trachomatis*, *Mycobacterium*, *Bartonella*, and *Coxiella* must contend with nutrient limitation imposed by the vacuolar membrane (Santos and Enninga, 2016). To circumvent this, pathogens actively manipulate vesicular trafficking, inducing fusion of their vacuoles with nutrient-rich organelles like the Golgi apparatus, ER-derived vesicles, or autophagosomes. For instance, *Legionella* forms a replicative vacuole that avoids lysosomal fusion and instead recruits ER-derived membranes through the action of type IV secretion system effectors (Ham et al., 2011; Kellermann et al., 2021). *C. trachomatis* similarly relies on exocytic vesicle transport (Eisenreich et al., 2019).

In contrast, cytosolic pathogens like *Listeria monocytogenes*, *Shigella flexneri*, and *Rickettsia* spp. have direct access to host cytosolic metabolites but must evade immune detection and oxidative stress (Bierne et al., 2018; O’Riordan and Portnoy, 2002). These pathogens often suppress or co-opt host immune signaling pathways. *Listeria*, for example, upregulates host antioxidant systems to counteract reactive oxygen species generated during infection (Chen et al., 2017; Ray et al., 2009). Additionally, these bacteria exhibit enhanced adaptability in carbon utilization, optimizing nutrient assimilation through streamlined transport and metabolic networks.

Despite opposing constraints guiding preference for vacuolar or cytosolic residence, growing evidence indicates these are not as exclusive as previously thought, with pathogens adapting alternative strategies based on host cell type and environmental cues (Petit and Lebreton, 2022).

### 3.2 Host-derived nutrient allocation and bipartite metabolism

Several intracellular bacterial pathogens employ a bipartite metabolic strategy, defined by the segregation of nutrient utilization into distinct functional networks dedicated to either energy production or biosynthetic processes (Best and Abu Kwaik, 2019). For energy generation, intracellular pathogens primarily catabolize host-derived C3 substrates such as pyruvate, glycerol, serine, and cysteine, which are abundant and relatively non-essential to host central metabolism (Eisenreich et al., 2015, 2019). These substrates are oxidized through the tricarboxylic acid (TCA) cycle, supporting ATP production via oxidative phosphorylation (OXPHOS) or substrate-level phosphorylation. Simultaneously, host glucose, glucose-6-phosphate, and related carbohydrates are preferentially allocated to anabolic pathways that support nucleotide synthesis, peptidoglycan assembly, and lipid biosynthesis (Table 2).

**TABLE 2:** Bipartite metabolic strategies of intracellular bacterial pathogens.

Bacterium	Intracellular niche	Energy source (catabolism)	Biosynthetic source (anabolism)	Key adaptations	Regulation	References
*L. monocytogenes*	Cytosol	Glycerol → glycolysis	Glucose-6-phosphate → PPP	Hpt transporter, lipoate scavenging	PrfA activation by BCAAs/glutamine	Freeman et al., 2025
*L. pneumophila*	LCV	Serine → TCA cycle	Glucose → PHB granules	Dot/Icm effectors, SLC1A5 recruitment	(p)ppGpp stringent response	Oliva et al., 2018
*C. trachomatis*	Inclusion	Malate → TCA cycle	Glucose-1-phosphate → glycogen	Inc proteins, Trp salvage from microbiota	Developmental cycle control	N’Gadjaga et al., 2022
*M. tuberculosis*	Phagosome	Fatty acids →β-oxidation	Host cholesterol → membrane lipids	ICL, Mce transporters	DosR regulon (hypoxia)	Forrellad et al., 2013
*S.* Typhimurium	SCV	Arginine → proline pathway	Gluconate → nucleotides	SPI-2 effectors, ArgT transporter	PhoP/PhoQ two-component	Groisman, 2001
*B. abortus*	rBCV	Erythritol → TCA cycle	Host glutamine → purines	BvrR/BvrS system, T4SS	CtrA cell cycle regulator	Rivas-Solano et al., 2022

LCV, *Legionella*-containing vacuole; SCV, *Salmonella*-containing vacuole; rBCV, replicative *Brucella*-containing vacuole; PPP, pentose phosphate pathway; PHB, poly-3-hydroxybutyrate; ICL, isocitrate lyase; T4SS, type IV secretion system.

This metabolic partitioning allows pathogens to sustain replication while minimizing competition with host metabolism. It also provides flexibility to adapt to nutrient fluctuations and governs phase transitions between replication and persistence. Critically, virulence gene expression is often repressed in glucose-rich environments but activated during amino acid-based metabolism, underscoring a direct connection between metabolic state and pathogenicity.

Bipartite metabolic strategies have been characterized in diverse intracellular pathogens. *L. pneumophila* predominantly catabolizes amino acids, especially serine, during intracellular replication, and transitions to glucose metabolism and polyhydroxybutyrate (PHB) accumulation during the transmissive phase (George et al., 1980; Price et al., 2011; Table 2). *C. burnetii* similarly relies on serine as an energy source while utilizing glycerol for anabolic biosynthetic needs. *Listeria monocytogenes* channels host-derived glycerol into energy-generating pathways, reserving glucose-6-phosphate for biosynthetic activities via the pentose phosphate pathway (Chen et al., 2017). *C. trachomatis* metabolizes host malate to fuel carbon flux through the TCA cycle while coordinating glycogen storage and degradation to support distinct stages of its developmental cycle (Gehre et al., 2016; N’Gadjaga et al., 2022; Table 2).

Thus, bipartite metabolism reflects a highly specialized adaptation that integrates nutrient sensing, metabolic reprogramming, and virulence control within the constrained intracellular environment of the host. These partitioned metabolic strategies not only enable intracellular survival but also set the stage for pathogen-specific adaptations to diverse host environments.

## 4 Pathogen-specific intracellular metabolic strategies expanded

These brief examples underscore the complexity and diversity of metabolic strategies among intracellular pathogens. Each species has evolved specialized systems for nutrient acquisition, metabolic regulation, and immune evasion, tailored to its specific intracellular niche. Understanding these pathogen-specific metabolic signatures provides a powerful framework for developing targeted antimicrobial therapies and identifying biomarkers of infection.

### 4.1 Listeria monocytogenes

The intracellular survival of *Listeria monocytogenes* depends on dynamic metabolic reprogramming of both bacterial and host pathways (Table 2). After entering macrophages or epithelial cells, *L. monocytogenes* secretes listeriolysin-O (LLO) and phospholipase C to rupture the phagosome and access the nutrient-rich cytosol, under regulation by the master virulence activator PrfA (Coelho et al., 2019; Lam et al., 2011).

In the cytosol, *Listeria* shifts to host-derived glycerol catabolism, upregulating glycerol kinase and glycerol-3-phosphate dehydrogenase for ATP production (Chen et al., 2017; Fuchs et al., 2012). Glucose-6-phosphate imported from the host pentose phosphate pathway supports nucleotide and cell wall biosynthesis while generating NADPH for oxidative stress resistance (Table 1). Host glutamine promotes proliferation and activates PrfA, while branched-chain amino acid sensing through CodY regulates virulence gene expression (Joseph et al., 2008; Lobel and Herskovits, 2016; Lobel et al., 2015). Lipoic acid scavenging sustains pyruvate dehydrogenase activity and acetyl-CoA production (Christensen et al., 2011; Sauer et al., 2019).

Beyond its metabolism, *L. monocytogenes* manipulates host mitochondrial function. Infection downregulates mitochondrial ribosomal protein Mrps35, impairing mitochondrial protein synthesis and oxidative metabolism, likely reducing host defenses (Yuan et al., 2021). Compensatory upregulation of the cytosolic ribosomal protein Rpl22l1 reflects reprogramming of host translation, an adaptation dependent on LLO activity (Yuan et al., 2021).

As infection progresses, *L. monocytogenes* downregulates ActA expression, halts actin polymerization, and becomes sequestered into lysosome-like *Listeria*-containing vacuoles (LisCVs) (Kortebi et al., 2017). Within these compartments, bacterial replication slows, and some subpopulations enter a viable but non-culturable (VBNC) state, maintaining membrane integrity despite loss of culturability. Dormant bacteria can reactivate and resume cytosolic motility when conditions permit. *Listeria* also exhibits persistence in Spacious *Listeria*-containing Phagosomes (SLAPs), particularly within macrophages (Kortebi et al., 2017). SLAPs represent an earlier-described vacuolar niche where bacteria avoid degradation while maintaining minimal metabolic activity.

To survive intracellular stresses, *L. monocytogenes* utilizes the LisRK two-component system, regulating genes critical for acid, ethanol, and osmotic stress resistance (Alejandro-Navarreto and Freitag, 2024). Activation of LisRK supports adaptation to hostile intracellular environments, complementing the metabolic remodeling required during the transition from cytosolic growth to vacuolar persistence.

### 4.2 Legionella pneumophila

*Legionella pneumophila* displays bipartite metabolism and biphasic regulation through its intracellular lifecycle (Table 2). Within the *Legionella*-containing vacuole (LCV), *L. pneumophila* enters a replicative phase, fueled by host-derived amino acids such as serine and cysteine, which feed into the TCA cycle for energy generation (Oliva et al., 2018). Nutrient cues trigger the transition to a transmissive phase, characterized by metabolic downshifts and increased virulence. To facilitate intracellular survival, *L. pneumophila* manipulates host metabolism through its Dot/Icm type IV secretion system, injecting over 300 effectors (Price et al., 2011). These include AnkB, which promotes proteasomal degradation of host proteins to liberate amino acids, and LpSpl, which disrupts host sphingolipid metabolism to inhibit autophagy. Host-derived solute carriers (SLCs) may also be recruited to the LCV membrane to enable nutrient uptake (Best and Abu Kwaik, 2019; Best et al., 2018).

During nutrient-rich conditions, *L. pneumophila* prioritizes amino acid catabolism. However, as amino acids become scarce, the bacterium switches to glucose metabolism through the Entner-Doudoroff (ED) pathway (Fonseca and Swanson, 2014; Harada et al., 2010). Glucose catabolism supports the synthesis of poly-3-hydroxybutyrate (PHB) for long-term energy storage, a critical adaptation for environmental survival and transmission (Best and Abu Kwaik, 2019). This metabolic shift is linked to the transition into the post-exponential phase, during which *L. pneumophila* becomes more cytotoxic, motile, and osmotically resistant (Oliva et al., 2018).

Complex regulatory cascades govern these transitions, with the bacterial alarmone (p)ppGpp acting as a central mediator. Upon amino acid depletion, uncharged tRNAs activate RelA, triggering ppGpp synthesis, which in turn activates RpoS and multiple two-component systems to induce stress resistance, motility, and virulence factors (Hammer and Swanson, 1999; Richards et al., 2013; Srivatsan and Wang, 2008). Further enhancing intracellular survival, *L. pneumophila* reprograms host mitochondria through the MitF effector, promoting mitochondrial fragmentation and a Warburg-like metabolic state that favors bacterial replication (Eisenreich et al., 2019; Escoll et al., 2017). Additionally, *L. pneumophila* demonstrates metabolic flexibility by utilizing host pyruvate and ketone bodies under nutrient-limited conditions and modulating host phosphoinositide trafficking to enrich the LCV with lipids and carbohydrates (Manske and Hilbi, 2014; Rolando et al., 2016).

### 4.3 Shigella flexneri

*Shigella* employs a type III secretion system to induce uptake of the bacterium into a vacuole, which is then lysed, and bacterial replication occurs within the host cytoplasm (Pieper et al., 2013). Tinevez et al. (2019) pose that *Shigella* actively depletes oxygen during tissue colonization, and the formation of hypoxic foci represents a characteristic adaptive strategy among enteropathogenic bacteria. Throughout uptake, replication, and spread, *Shigella* virulence factors remodel the host cytoskeleton by altering carbon metabolism and transport pathways (Lu et al., 2024; Waligora et al., 2014).

Ferrari et al. (2019) showed that effectors LpaJ and VirA block host cell secretion, impair receptor recycling, and reduce receptor-mediated endocytosis. They found IpaJ inhibited STING activation of the IFN pathway by preventing STING translocation from the endoplasmic reticulum (ER) to ER-Golgi intermediate compartment (ERGIC). Meanwhile, VirA was reported to impair host cell secretory transport and inhibit autophagy by acting as a Rab-GTP activating protein (GAP). Kentner et al. (2014) observed infected cells maintained stable energy production despite the metabolic burden imposed by rapid *Shigella* reproduction. *Shigella* likely redirects many host metabolites, such as acetate and pyruvate, that would normally be excreted toward fermentation pathways (Kentner et al., 2014).

*Shigella* employs several strategies to deal with the stress of shifting environmental pressures as it changes residence from the outside the epithelial cell to the intravacuolar and extracytosolic compartments. Specialized adaptations in polyamine metabolism- particularly SpeG inactivation leading to increased use of putrescine and spermidine contribute to cytosolic survival and DNA stabilization during oxidative stress (Barbagallo et al., 2011). In response to changing oxygen tension, iron acquisition is also tightly regulated, with different expression pathways activated under aerobic and anaerobic conditions (Wyckoff et al., 2009).

### 4.4 Chlamydia trachomatis

The type III secretion system (T3SS) of *Chlamydia trachomatis* delivers effector proteins into the host cytosol and inclusion lumen to manipulate host metabolism. Effectors such as TarP and TepP modulate actin dynamics, activate PI3K/Akt signaling, and inhibit immune responses triggered by TLR2, NOD1, and STING (Chen et al., 2014; Elwell et al., 2016; Jury et al., 2023). TepP-mediated recruitment of PI3K facilitates PIP3 synthesis, enhancing early inclusion formation and vesicle trafficking. CpoS, another critical effector, suppresses host cell death pathways and regulates Rab GTPases, maintaining inclusion integrity and promoting survival (Meier et al., 2023; Stelzner et al., 2023). Downregulation of p53 and remodeling of mitochondrial architecture may inhibit apoptosis during rapid bacterial replication and serve as a source of glycolytic intermediates (Rother et al., 2019). Inclusion membrane proteins (Incs) such as IncA, IncG, and IncD facilitate vesicle fusion, nutrient uptake, and preservation of the replicative niche (Mital et al., 2013; Weber et al., 2016).

*Chlamydia* secures host-derived nutrients by hijacking Golgi- and ER-derived vesicles. Host transporters like SLC35D2 and Glut1 are redirected to import glucose and glucose-1-phosphate, fueling glycogen synthesis within the inclusion (Wang et al., 2017). Stored glycogen is metabolized during the reticulate body (RB) stage to support ATP production and nucleotide biosynthesis, exemplifying the bipartite metabolic strategy wherein host-derived nutrients are selectively partitioned for energy and anabolic processes (Colpaert et al., 2021; Omsland et al., 2014). Lipid metabolism is similarly reprogrammed: *C. trachomatis* captures ceramide and phosphatidic acid for inclusion membrane biogenesis and modifies cholesterol to stabilize inclusion structure and dampen immune activation (Yao and Rock, 2018).

To bypass tryptophan depletion mediated by IFN-γ, *C. trachomatis* expresses functional tryptophan synthase (TrpA), utilizing indole from the microbiota to continue protein synthesis during immune stress (Wang et al., 2022; Xie et al., 2002). Metabolic profiling of infected cells reveals a shift toward a Warburg-like state (Rother et al., 2019), with increased pyruvate, lactate, and glutamate levels, favoring glycolysis over oxidative phosphorylation. This host metabolic reprogramming enhances the availability of anabolic intermediates, facilitating rapid bacterial replication. Deubiquitinating enzymes like ChlaDUB1 further support infection by stabilizing anti-apoptotic proteins and glucose transporters (Jury et al., 2023).

Under nutrient deprivation, cytokine exposure, or antibiotic pressure, *C. trachomatis* reticulate bodies differentiate into enlarged, persistent aberrant bodies (ABs) (Panzetta et al., 2018). These non-dividing forms reduce metabolic activity but maintain viability, allowing reversion to active replication once favorable conditions are restored. This transition to persistence reflects another layer of metabolic flexibility critical for *C. trachomatis* survival during chronic or latent infections.

## 5 Metabolism and antimicrobial resistance

The intracellular niche represents a privileged environment that shields pathogens from antibiotics. Reaching infected cells presents an initial hurdle that increases the time required for antibiotics to encounter pathogens, and the effective dose necessary (Jo, 2019). Alterations in host cell metabolism affect the ability of antibiotics to be taken up by cells, reach the intracellular location of a pathogen, and cause them to be quickly altered or extruded (Eisenreich et al., 2019). In addition to altering host metabolism, intracellular pathogens have unique mechanisms to regulate their individual and collective metabolism, including adjusting cell wall processing and expression of efflux pumps. In contrast to extracellular pathogens, the intracellular bacteria may be primed to adapt to rapid physiological shifts such as those occurring during phagocytosis and lysosomal escape. This involves differences in phenotypic plasticity, gene expression mechanisms, and proclivity for lifecycle changes. Switching between rapid growth and slower maturation or latent phases, in particular, can contribute to deflecting antibiotic targeting.

Albeit a facultative intracellular bacterium, *S. aureus* is one of the most extensively studied pathogens, offering broadly applicable insights into intracellular persistence mechanisms. Studies have shown that many antibiotics poorly penetrate *S. aureus* infected neutrophils (Bongers et al., 2019). For instance, aminoglycosides are barred from entering due to high polarity and mainly accumulate in lysosomes, where their action is inhibited by low pH. Tetracycline uptake is moderate; however, it occurs very slowly, possibly relying on a cation transport mechanism. While Imipenem can rapidly accumulate in neutrophils, it does not reach considerable concentrations, potentially due to the compound being swiftly altered or metabolized. Intracellular *S. aureus* persisters exhibit biphasic killing dynamics and enter a non-dividing, antibiotic-tolerant state when examined at the single-cell level (Peyrusson et al., 2020). These remain metabolically active and display a distinct transcriptomic profile marked by the upregulation of multiple stress responses, including the stringent response, cell wall stress response, SOS response, and heat shock response, which are reversible upon removal of antibiotic stress. Furthermore, these persisters demonstrated higher multidrug resistance (MDR) upon subsequent challenge.

Among environmental pathogens, acidic conditions have been shown to reduce lateral gene transfer and have a bactericidal effect (Yu et al., 2023). Highly specialized intracellular pathogens adapted to human hosts, however, may take advantage of the rapid pH shift occurring on cell entry to enact another obstacle to antimicrobial access. Some studies suggest the spatial constraint imposed by vacuoles may enhance lateral gene transfer and quorum sensing among bacterial microcolonies (Personnic et al., 2019). Antibiotics may not reach uniform distribution within the host cell or sub-compartments, and differences in exposure may contribute to the effect of lag times in bacterial subpopulations on resistance emergence (Camacho Mateu et al., 2021). Computational tools are currently being developed to help explore such “adaptive dynamics.” Additionally, pleiotropic effects associated with lifestyle changes of intracellular pathogens may alter fitness effects of mutations that confer AMR (Matange et al., 2019). For instance, in *M.tb*, iron starvation triggers the isoniazid-induced gene operon *iniBAC* that may subsequently be involved in resistance (Gupta et al., 2023).

An interesting point for further research concerns bacterial detection of antimicrobial compounds through mechanisms similar to those we have discussed regarding metabolic shifts coordinated with nutrient sensing. The question of pathogens’ ability to detect distal compounds or environmental stress has been posed in studies on quorum sensing, but is not well understood. Bacterial cell morphology is also thought to influence antimicrobial resistance, with surface to surface-to-volume ratio being a likely factor that is not well characterized (Ojkic et al., 2022). Compared to extracellular pathogens, knowledge on morphologic changes during the life of intracellular pathogens is lacking- in part due to the difficulty of reproducing infection conditions *in vitro*.

## 6 Metabolic constraints and fitness trade-offs in intracellular pathogens

While a range of mechanisms is employed for bacteria to overcome adverse conditions, including adaptations and phenotypic plasticity, successful intracellular pathogens become very specialized, considering the greater genetic diversity among similar species. Over evolutionary time, prolonged host association has driven many pathogens to shed genes for redundant metabolic pathways, resulting in genome reduction and increased host dependence; a trend toward auxotrophy (Castelli et al., 2024).

Recent genomic analyses highlight this trend (Medicielo et al., 2023). For example, *Rickettsia* has lost up to 20 metabolic pathways, including terpenoid biosynthesis, compared to its vacuole-residing relative *Anaplasma*; likely as a consequence of adapting to the host cytosol. Similarly, *M. tuberculosis*, the only obligate intracellular pathogen within the *Mycobacterium* complex, exhibits substantial genome reduction relative to *M. canettii*, a more environmentally adapted and genetically heterogeneous relative (Fabre et al., 2017; Sous et al., 2024; Supply and Brosch, 2017). These patterns suggest that intracellular specialization involves both metabolic dependency and constrained genetic flexibility.

Conversely, *L. pneumophila* retains a broader metabolic repertoire and shows high conservation of core virulence traits across the genus (Wexler et al., 2022). Its preference for amino acid catabolism over glucose—unusual among many bacteria, may reflect adaptation to the nutrient environment within protist hosts, where digestion of engulfed prey can generate amino-acid–rich conditions (Best and Abu Kwaik, 2018, 2019). Reliance on host-derived amino acids may serve as a growth-limiting strategy outside of host cells, reinforcing intracellular dependence.

Horizontal gene transfer has further shaped *L. pneumophila*’s metabolic landscape (de Felipe et al., 2005). Its genome encodes numerous eukaryotic-like proteins, including 11 solute carrier (SLC)-like transporters with strong structural homology to host nutrient transporters (Best et al., 2018). These proteins are thought to facilitate nutrient acquisition from the host cytosol, reinforcing *Legionella*’s capacity to thrive within eukaryotic cells.

Commensal bacteria and pathogens have been shown to benefit from the presence of neighboring bacteria through various interbacterial signaling methods, nutrient scavenging, and other communal behaviors (Garcia-Santamarina et al., 2024). In this context, achieving an isolated [lone] niche represents another fitness tradeoff for intracellular bacteria and might be interesting to analyze from a systems perspective.

## 7 Applications of host-directed therapies in treating intracellular pathogens

The poor bioavailability of antibiotics in infected tissues often necessitates higher dosing and prolonged treatment regimens, particularly when combating drug-resistant infections. Host-directed therapies (HDTs) represent a promising adjunct or alternative strategy, as they aim to enhance the host’s intrinsic ability to control infection rather than acting directly on the pathogen (Gholap et al., 2025; van den Biggelaar et al., 2024; Table 3). Because HDTs primarily target host pathways rather than bacterial components, they are generally thought to impose less direct selective pressure than conventional antibiotics and may therefore be less likely to drive antimicrobial resistance. When used in combination with antimicrobials, HDTs may offer synergistic benefits, lower the required dose of conventional drugs and improving efficacy against resistant strains.

**TABLE 3:** Targeting pathogen-exploited host metabolism via host-directed therapies (HDTs).

Metabolic pathway	Pathogen exploitation	HDT strategy	Example agents	Mechanism of action	References
Lipid metabolism	*M. tuberculosis* scavenges host cholesterol via Mce4 transporters	Inhibit host cholesterol synthesis	Atorvastatin, rosiglitazone	↓ HMG-CoA reductase (statins) or ↑ PPARγ-driven lipid oxidation	Roth et al., 2024
Iron homeostasis	*Legionella* secretes siderophores (legiobactin) to steal iron	Restrict host iron availability	Hepcidin mimetics (PTG-300), Iron chelators (deferasirox)	↓ Ferroportin-mediated iron export or sequester free iron	Cianciotto, 2015
TCA cycle	*Chlamydia* depletes α-ketoglutarate (α-KG), impairing host epigenetics	Supplement TCA intermediates	α-KG esters, DMF (dimethyl fumarate)	Restore α-KG-dependent histone demethylation	Pennini et al., 2010
Amino acid auxotrophy	*Listeria* depends on host BCAAs for CodY-mediated virulence	Starve pathogens of amino acids	BCAA mimetics (leucine analogs)	Competitive inhibition of bacterial BCAA transporters	Brenner et al., 2018
Tryptophan scarcity	*C. trachomatis* uses microbiota-derived indole to bypass IFN-γ-induced starvation	Block tryptophan salvage	Indole analogs, IDO inhibitors (epacadostat)	Inhibit bacterial tryptophan synthase or host IDO	Wang et al., 2022

↑ Indicates increase/upregulation and ↓ indicates decrease/downregulation.

Unlike traditional antimicrobials, many HDTs function by modulating host cell signaling pathways (Shapira et al., 2024), enhancing antimicrobial functions such as autophagy, inflammation regulation, or phagocyte activation. Several FDA-approved drugs have already been repurposed as HDTs, including metformin, aspirin, and glibenclamide. The celecoxib derivative AR-12 (OSU-03012) has shown particular promise, significantly reducing *S. enterica* serovar Typhimurium burden and improving post-infection survival in murine models (Varma et al., 2020). AR-12 has also demonstrated efficacy against *F. tularensis* through an autophagy-mediated mechanism, highlighting its potential for broad-spectrum host-directed applications (Table 3).

Efficient delivery remains a major focus of HDT advancement. Drug-loaded micro- and nanoparticle systems have proven effective in protecting therapeutic cargo from degradation while extending systemic circulation time (Varma et al., 2020). These platforms can be engineered with customizable size, charge, and surface properties to enhance uptake, cellular targeting, and controlled release. Advances in computational modeling have improved the design of such particles for tissue-specific delivery, including emerging intranasal and intratracheal routes that may benefit treatment of respiratory infections like tuberculosis.

Despite growing interest, many HDT studies remain in the exploratory phase, and optimization through structure-activity relationship (SAR) studies is still lacking. A recent systematic review by Shapira et al. (2024) emphasized that while HDTs show the greatest promise when combined with antimicrobials, the field lacks comprehensive investigation into key host pathways. For example, cell cycle regulation remains an underexplored target, with only two compounds identified to date. Furthermore, the assumption that HDTs are “resistance-proof” requires more robust experimental validation.

## 8 Conclusion

The intracellular lifestyle imposes extreme metabolic constraints on bacterial pathogens, requiring the evolution of nuanced and multifaceted strategies to thrive within hostile host environments. Through the lens of metabolomics and evolutionary biology, this review has examined how pathogens engage in an immunometabolic dialog with host cells, coordinating metabolic rewiring to navigate nutrient scarcity, immune activation, and environmental stress. These strategies are neither isolated nor static—they reflect an integrated, dynamic response to intracellular niche, infection stage, and host immune status.

Across diverse organisms such as *Listeria monocytogenes*, *Salmonella enterica*, *Mycobacterium tuberculosis*, *Legionella pneumophila*, *Chlamydia trachomatis*, and *Shigella flexneri*, common themes emerge: the co-option of host nutrient pathways, the tuning of carbon source preferences according to intracellular location, and the strategic suppression or activation of metabolic networks to transition between growth and persistence. Regulatory hubs such as PrfA, DosR, PhoP/PhoQ, and CodY orchestrate these metabolic shifts in response to cues like oxygen tension, nutrient gradients, and redox stress.

Bipartite metabolism, in which intracellular pathogens partition nutrient use between energy production and biosynthesis, appears to be a broadly conserved strategy across species. This division is tightly coupled to global transcriptional programs, post-translational control, and feedback from host-derived metabolic signals. Within this framework, metabolic flexibility supports both persistence and virulence. From a translational standpoint, such metabolic architectures expose vulnerabilities. Targeting pathogen-specific transporters, regulatory hubs, or host-pathogen nutrient interfaces may offer a rational basis for precision antimicrobial development.
